# The impact of body mass index on treatment response to high-dose dexamethasone in adult primary immune thrombocytopenia patients: A two-center retrospective study

**DOI:** 10.1007/s11739-026-04318-w

**Published:** 2026-03-13

**Authors:** Abdulkerim Yıldız, Rafiye Çiftçiler, Samet Yaman, Minura Abishova Aliyeva, Sedat Acar, Shabnam Shahab, Huriye Baysal

**Affiliations:** 1 Department of Hematology, Faculty of Medicine, Erol Olçok Training and Research Hospital, Hitit University, Çorum, Turkey; 2 Division of Hematology, Department of Internal Medicine, Faculty of Medicine, Selçuk University, Konya, Turkey; 3 Department of Hematology, Melhem International Hospital, Baku, Azerbaijan; 4 Department of Internal Medicine, Faculty of Medicine, Erol Olçok Training and Research Hospital, Hitit University, Çorum, Turkey; 5 Department of Internal Medicine, Faculty of Medicine, Selçuk University, Konya, Türkiye

**Keywords:** Dexamethasone, Body mass index, Immune thrombocytopenia, Response

## Abstract

High-dose dexamethasone (HDD) is widely used as first-line therapy for immune thrombocytopenia (ITP) and is administered at a fixed dose regardless of body weight. The impact of body mass index (BMI) on treatment response to HDD remains unclear. This retrospective, two-center study included 60 adult patients with newly diagnosed ITP who received HDD as first-line therapy. Demographic characteristics, BMI, baseline laboratory values, and treatment responses at 1, 6, and 12 months were analyzed. BMI was evaluated using cut-off values of 25, 27, and 30. The median number of HDD cycles administered was 1 (range: 1–4), and the median BMI at diagnosis was 27.0 kg/m^2^ (range: 18.0–44.0). No significant differences were observed between BMI categories with regard to treatment responses at months 1 and 6 (*p* > 0.05 for both). However, at month 12, a complete response (CR) was more likely in patients with BMI < 30, and a partial response (PR) in those with BMI ≥ 30 (*p* = 0.023). Across all time points, no other demographic or clinical variable emerged as an independent predictor of treatment response (*p* > 0.05). The results of this study indicate that in newly diagnosed ITP patients receiving HDD as first-line treatment, BMI does not influence early or durable treatment responses, although it may have a modest adverse effect on late response. Larger prospective studies are needed to clarify underlying mechanisms and assess whether obesity-related factors should inform individualized treatment.

## Introduction

Immune thrombocytopenia (ITP) is an acquired autoimmune disorder characterized by isolated thrombocytopenia resulting from immune-mediated platelet destruction and impaired platelet production [1]. In addition to prednisolone, which has long been the standard treatment for ITP, the alternative agent included in the guidelines is high-dose dexamethasone, which is used at the standard dose regardless of the patient's weight, unlike prednisolone treatment. The guidelines recommend oral high-dose dexamethasone (HDD) at a fixed dose of 40 mg daily for 4 consecutive days [2, 3]. Apart from corticosteroids, available treatment options for ITP include thrombopoietin receptor agonists, rituximab, and newer immunosuppressive agents such as fostamatinib, particularly in patients with relapsed or refractory disease [1].

Obesity may theoretically influence drug distribution, metabolism, and overall pharmacological activity [4, 5]. Obesity is known to alter glucocorticoid pharmacokinetics, but the data evaluating its impact on HDD response in ITP are limited and inconclusive. Given the increasing prevalence of obesity worldwide, understanding whether BMI affects treatment response is clinically relevant. The aim of this study was to evaluate patient characteristics, BMI distributions, and treatment outcomes across multiple BMI thresholds to determine whether BMI has an effect on early or sustained response to HDD in newly diagnosed adult ITP patients.

## Methods

This two-center, retrospective study was conducted with adult patients diagnosed with primary ITP who received HDD (40 mg/day for 4 days) as first-line therapy in the 6-year period of January 2019 to January 2025. The patients included in the study were aged ≥ 18 years, newly diagnosed, treated with steroids as first-line therapy, and followed up for at least 12 months after starting treatment. Patients diagnosed with ITP who did not require treatment were excluded from the study. Patients with missing key clinical data were also excluded. The diagnostic criteria for ITP were based on the international consensus of ITP [6].

HDD was selected as first-line therapy in accordance with international guidelines and institutional practice, as it enables rapid platelet recovery with a short treatment duration while minimizing prolonged corticosteroid exposure associated with prednisone. The decision to initiate HDD or prednisolone was left to the discretion of the treating clinicians, as no predefined treatment protocol was mandated; treatment selection was based solely on individual clinical judgment and experience. Accordingly, our study included newly diagnosed ITP patients who received HDD as first-line therapy according to clinician preference. The number of cycles of HDD treatment for each patient was determined by the clinicians.

Intravenous immunoglobulin (IVIG) use was recorded when administered. IVIG was given as a single treatment course at diagnosis at a dose of 0.4 g/kg/day for 3–5 days, depending on bleeding status. Patients who were refractory to HDD or who initially responded but subsequently relapsed and required second-line therapy were excluded from subsequent response assessment endpoints to avoid confounding effects from additional treatment lines. During the study period, no patients received thrombopoietin receptor agonists, rituximab, or other immunosuppressive agents; treatment consisted exclusively of HDD with or without IVIG.

Age, sex, number of comorbidities, BMI, baseline platelet count, biochemical test results, and vitamin B12 and ferritin levels were recorded. Treatment response was assessed at 1, 6, and 12 months after initiation of first-line therapy and defined as complete response (CR): platelet count ≥ 100 × 10^9^/L and absence of bleeding; partial response (PR): platelet count ≥ 30 × 10^9^/L and at least doubling of the baseline count, and absence of bleeding; no response (NR): platelet count < 30 × 10^9^/L or less than doubling of the baseline count, or bleeding [7].

Statistical analyses were conducted using IBM SPSS Statistics vn 27 software. Frequency tables and descriptive statistics were used to interpret the findings. Conformity of the data to normal distribution was assessed. The measurements of two independent groups of data showing normal distribution were compared using the Independent Samples *t* test (*t*-table value), and for measurements not conforming to normal distribution, the Mann–Whitney *U* test (*Z*-table value) was applied. Pearson-*χ*2 crosstabs were used to examine the relationships between two qualitative variables. A value of *p* ≤ 0.05 was accepted as statistically significant.

### Compliance with ethical standards

All procedures performed in studies involving human participants were in accordance with the ethical standards of the institutional and/or national research committee and with the 1964 Helsinki Declaration and its later amendments or comparable ethical standards. Approval for this study was granted by Hitit University Ethics Committee (approval number: 2025–206 dated: 04/11/2025).

## Results

A total of 60 patients (42 female, 18 male) were included in the study. The median number of dexamethasone cycles administered was 1 (range: 1–4). The median BMI at diagnosis was 27.0 kg/m^2^ (range: 18.0–44.0). The demographic characteristics of the study population are presented in Table 1. BMI categories were defined using cut-off values of 25 and 30 according to WHO recommendations [8]. Given that the median BMI of the cohort was 27, an additional cut-off value of 27 was also applied to further categorize the patient groups. Demographic and clinical characteristics were compared among the groups. From these categories, the number of comorbidities was significantly higher in patients with a BMI ≥ 30 compared with those with a BMI < 30 (*p* = 0.038). Initial ferritin levels were higher in patients with a BMI ≥ 25 compared with those with a BMI < 25 (*p* = 0.029). However, ferritin levels at diagnosis did not differ significantly among the other BMI categories. All other demographic and clinical parameters were similar across all the groups. Detailed comparisons of the demographic and clinical characteristics according to BMI categories are shown in Table 2. Patients were then evaluated in terms of their response to dexamethasone treatment. No significant differences were observed between the BMI groups regarding treatment response at months 1 and 6 (*p* > 0.05 for both). However, at month 12, patients with a BMI < 30 were more likely to achieve a complete response (CR), whereas those with a BMI ≥ 30 were more likely to achieve a partial response (PR) (*p* = 0.023). The treatment response comparisons according to BMI categories are summarized in Table 3. IVIG use was similar in patients with BMI below 30 and those with BMI above 30 (43.5% vs 42.9%; *p* = 0.967).

**Table 1:** Demographic and clinical characteristics of patients

(*N* = 60)	%
Gender (*n*,%)	
Female Male	42 (70) 18 (30)
Age at diagnosis (years) X ® ± SD	52.42 ± 18.99
Number of comorbidities (median, [min–max])	1.0 [0.0–7.0]
Number of HDD cycles (median, [min–max])	1.0 [1.0–4.0]
Platelet count at diagnosis (× 10^6^/l (median, [min–max])	10,500.0 [1000.0–74000.0]
BMI at diagnosis (median, [min–max])	27.0 [18.0–44.0]
IVIG usage (*n*,%)	
Yes No	26 (43.3) 34 (56.7)

*SD* standard deviation, *BMI* body mass index, *IVIG* intravenous immunoglobulin, *HDD* high-dose dexamethasone

**Table 2:** Comparisons of demographic and clinical parameters according to BMI category

Parameter	BMI < 25.0 (*n* = 16)	BMI ≥ 25.0 (*n* = 44)	*p*	BMI < 27.0 (*n* = 30)	BMI ≥ 27 (*n* = 30)	*p*	BMI < 30.0 (*n* = 46)	BMI ≥ 30.0 (*n* = 14)	*p*
Gender (*n*,%)									
Female Male	12 (75.0%) 4 (25.0%)	30 (68.2%) 14 (31.8%)	0.610	21 (70.0%) 9 (30.0%)	21 (70.0%) 9 (30.0%)	1.000	32 (69.6%) 14 (30.4%)	10 (71.4%) 4 (28.6%)	0.894
IVIG usage (*n*,%)									
Yes No	6 (37.5%) 10 (62.5%)	20 (45.5%) 24 (54.5%)	0.582	14 (46.7%) 16 (53.3%)	12 (40.0%) 18 (60.0%)	0.602	20 (43.5%) 26 (56.5%)	6 (42.9%) 8 (57.1%)	0.967
Age at diagnosis (years) X ® ± SD	44.43 ± 23.83	55.31 ± 16.26	0.107	48.93 ± 21.11	55.90 ± 16.23	0.157	50.84 ± 20.12	57.57 ± 14.06	0.250
Number of comorbidities (median IQR)	1.0 [2.5]	1.0 [1.8]	0.439	1.0 [1.3]	1.0 [1.0]	0.110	1.0 [2.0]	2.0 [1.5]	**0.038**
Number of HDD cycles (median IQR)	1.0 [1.8]	1.5 [2.0]	0.268	1.0 [2.0]	2.0 [3.0]	0.089	1.0 [2.0]	2.0 [3.0]	0.417
Platelet count at diagnosis (× 10^6^/l, median IQR)	15,000.0 [15750.0]	7000.0 [17000.0]	0.160	10,000.0 [15250.0]	13,000.0 [17500.0]	0.300	10,500.0 [17000.0]	12,500.0 [20750.0]	0.391
Ferritin (ng/ml)	33.5 [34.8]	53.0 [116.0]	**0.029**	35.0 [59.0]	53.0 [100.5]	0.176	37.5 [93.5]	48.0 [127.5]	0.262

Statistically significant results (*p* < 0.05) are indicated in bold

*BMI* body mass index, *IVIG* intravenous immunoglobulin, *HDD* high-dose dexamethasone

**Table 3:** Comparisons of treatment responses according to BMI category

Response at	BMI			BMI			BMI		
	< 25.0 (*n* = 16)	≥ 25.0 (*n* = 44)	*p*	< 27.0 (*n* = 30)	≥ 27 (*n* = 30)	*p*	< 30.0 (*n* = 46)	≥ 30.0 (*n* = 14)	*p*
1 month									
NR PR CR	4 (25.0%) 4 (25.0%) 8 (50.0%)	8 (18.2%) 11 (25.0%) 25 (56.8%)	0.830	9 (30.0%) 7 (23.3%) 14 (46.7%)	3 (10.0%) 8 (26.75) 19 (63.3%)	0.148	10 (21.7%) 11 (23.9%) 25 (54.4%)	2 (14.3%) 4 (28.6%) 8 (57.1%)	0.816
6 months									
NR PR CR	3 (25.0%) 2 (16.7%) 7 (58.3%)	6 (16.7%) 12 (33.3%) 18 (50.0%)	0.519	3 (14.3%) 5 (23.8%) 13 (61.9%)	6 (22.2%) 9 (33.3%) 12 (44.5%)	0.483	5 (13.9%) 9 (25.0%) 22 (61.1%)	4 (33.3%) 5 (41.7%) 3 (25.0%)	0.085
12 months									
NR PR CR	2 (22.2%) – 7 (77.8%)	3 (10.0%) 10 (33.3%) 17 (56.7%)	0.116	2 (11.1%) 3 (16.7%) 13 (72.2%)	3 (14.3%) 7 (33.3%) 11 (52.4%)	0.418	5 (16.1%) 5 (16.1%) 21 (67.8%)	– 5 (62.5%) 3 (37.5%)	**0.023**

Statistically significant results (*p* < 0.05) are indicated in bold

*BMI* body mass index, *CR* complete response, *PR* partial response, *NR* no response

The effects of clinical and demographic parameters other than BMI on the response to HDD treatment were evaluated. Patients were categorized into two groups according to treatment outcome: responders (CR + PR) and non-responders (NR). These groups were compared across selected demographic and clinical variables. Analysis of 1-month treatment responses revealed that responders had a significantly higher number of HDD courses compared with non-responders (*p* < 0.001). In addition, ferritin levels at diagnosis were significantly higher in 1-month non-responders than in responders (*p* = 0.027). However, no predictive factor for treatment response at 1 month was identified in the logistic regression analysis (*p* > 0.05). Evaluations of the 6-month and 12-month outcomes demonstrated no significant parameters associated with treatment response (*p* > 0.05 for both). The detailed analysis of factors influencing treatment response is presented in Table 4.

**Table 4:** Analysis of factors affecting response to treatment

	1-month response			6-month response			12-month response		
	CR + PR (*n* = 48)	NR (*n* = 12)	*p*	CR + PR (*n* = 39)	NR (*n* = 9)	*p*	CR + PR (*n* = 34)	NR (*n* = 5)	*p*
Gender									
Female Male	36 (75.0%) 12 (25.0%)	6 (50.0%) 6 (50.0%)	0.181	29 (74.4%) 10 (25.6%)	7 (77.8%) 2 (22.2%)	0.831	25 (73.5%) 9 (26.5%)	4 (80.0%) 1 (20.0%)	0.757
Age at diagnosis (years) X ® ± SD	51.29 ± 18.26	56.91 ± 21.94	0.363	51.43 ± 17.61	50.67 ± 22.06	0.911	50.47 ± 18.19	56.00 ± 12.42	0.379
Number of comorbidities (median IQR)	1.0 [2.0]	1.0 [3.0]	0.780	1.0 [2.0]	1.0 [2.0]	0.989	1.0 [2.0]	1.0 [2.0]	0.265
Platelet count at diagnosis (× 10^6^/l, median IQR)	12,500.0 [18750.0]	7500.0 [13000.0]	0.066	11,000.0 [18000.0]	14,000.0 [27000.0]	0.625	12,500.0 [20500.0]	11,000.0 [11500.0]	0.599
Number of HDD cycles (median IQR)	2.0 [3.0]	1.0 [0.0]	** < 0.001**	2.0 [3.0]	2.0 [2.0]	0.492	2.0 [3.0]	1.0 [3.0]	0.788
Blood group									
0 A AB B	7 (23.3%) 12 (40.0%) 2 (6.7%) 9 (30.0%)	3 (42.9%) 2 (28.6%) – 2 (28.6%)	0.690	6 (26.1%) 8 (34.8%) 2 (8.7%) 7 (30.4%)	1 (14.3%) 4 (57.1%) – 2 (28.6%)	0.658	6 (30.0%) 6 (30.0%) 1 (5.0%) 7 (35.0%)	– 2 (66.7%) 1 (33.3%) –	0.147
Vitamin B12 levels (pg/ml)	326.5 [173.5]	366.0 [216.5]	0.938	332.0 [173.0]	265.0 [285.5]	0.783	332.0 [155.0]	327.0 [881.5]	0.140
Ferritin (ng/ml)	35.0 [77.5]	112.0 [230.0]	**0.027**	36.0 [65.3]	35.0 [115.0]	0.742	38.5 [65.3]	28.5 [147.3]	0.831

Statistically significant results (*p* < 0.05) are indicated in bold

*CR* complete response, *PR* partial response, *NR* no response, *HDD* high-dose dexamethasone

## Discussion

ITP is a disease in which underlying immune mechanisms are crucial for both etiology and response to treatment [9]. Like the majority of all autoimmune diseases, ITP is an organ-specific disease and abnormalities in immune cell types, such as antigen-presenting cells (APC), T cells, and B cells, have been shown to play some sort of role in the initiation and/or perpetuation of the disease. Obesity is a condition that causes immune dysregulation, causes chronic inflammation in the body, and alters the pharmacokinetics of the drugs used [4, 10]. Therefore, there are important links between ITP and obesity, both in terms of causality and response to treatment. This issue has recently attracted the attention of researchers. In the current retrospective study, the influence of BMI on treatment response to fixed-dose HDD was evaluated in newly diagnosed adult ITP patients. Across multiple BMI cut-off values (< 27, < 30, < 25), BMI demonstrated no association with response rates at month 1 or month 6. These findings suggest that the dexamethasone fixed dosing regimen is effective across a wide range of body weight profiles. Although obesity may theoretically affect the pharmacokinetics of lipophilic corticosteroids, this did not translate into clinically meaningful differences in treatment outcomes in the current study cohort. Only in the BMI ≥ 30 group was a shift observed in month 12 response patterns, as these patients exhibited fewer CR and more PR. Importantly, the NR rate did not increase, indicating that obesity did not diminish overall treatment effectiveness.

Recent data have suggested that obesity may influence immune-related disorders, and the current study findings support this concept within the context of ITP. In a large retrospective cohort of treatment-naïve patients treated with HDD, obesity was consistently associated with poorer therapeutic outcomes [11]. Both overall and CR rates declined progressively with increasing BMI, with the obese subgroup exhibiting the lowest probability of achieving an early hematological response. This negative gradient persisted at 6 months, reflected by markedly reduced sustained response and sustained CR rates among obese patients. Importantly, multivariate analyses confirmed obesity as an independent predictor of inferior early and long-term treatment responses as well as increased relapse risk. In a previous cohort of 275 patients, stratified according to BMI, higher BMI was consistently associated with more unfavorable disease characteristics [12]. Obese patients presented with lower platelet counts both at diagnosis and at nadir, and demonstrated a greater need for therapeutic intervention as well as increased use of multiple treatment lines. Notably, among patients receiving corticosteroids, those with normal or lower BMI achieved longer remission durations and were less likely to develop steroid dependence compared with obese individuals. These observations suggest that obesity may not only predispose individuals to ITP but may also negatively influence its clinical course and treatment response. That study differs from the current study in several ways: There were 275 patients in that study, but 159 required treatment. Of those who did require treatment, approximately 60% received HDD. In other words, not all patients received HDD. A weight-adjusted treatment such as PDN was also used. Therefore, it would be more accurate to consider that study not in terms of the relationship between HDD and obesity, but rather as evidence for the hypothesis that obesity can negatively impact all stages of ITP. In the current study, only patients receiving HDD were included. As the use of HDD fixed dose eliminates weight adjustment, it allowed the direct effects of obesity on treatment response in ITP patients to be more rationally demonstrated. The most recent study on this subject is a retrospective cohort study by Everhardt T, et al. published in 2025 [13]. There were 49 patients who received a median of 1 course of HDD treatment, and responses were evaluated at 6 months. Obese patients with primary acute ITP were found to exhibit substantially lower initial and complete response rates compared with their nonobese counterparts, despite receiving standard HDD regimens. It was suggested that obesity-related factors, such as chronic low-grade inflammation, altered immune regulation, and possible changes in glucocorticoid pharmacokinetics, may attenuate steroid efficacy.

Based on the results of the studies mentioned above, obesity appears to generally reduce HDD responses in ITP patients. However, in the current study, it was found to have no effect on early and mid-range responses, with a slightly negative effect on late responses. This result demonstrates that different results are still possible in this area, and therefore highlights the need for larger, and if possible, randomized-controlled studies.

Factors affecting treatment response in ITP patients treated with steroids are not limited to BMI. Numerous studies have been conducted on this topic. One study showed that very early platelet response was predictive of treatment response only in patients receiving HDL [14]. The effects of demographic characteristics, vitamin B12, and ferritin levels were also examined in the current study, although not in detail. In our study, baseline ferritin levels were higher in patients with a BMI ≥ 25; however, no significant differences were observed across other BMI categories. Moreover, among patients with a BMI ≥ 30 who achieved a response at month 12, baseline ferritin levels were comparable to those of patients with lower BMI. These findings suggest that ferritin is unlikely to mediate the observed BMI-related differences in late treatment response, and that alternative obesity-related mechanisms may be involved. Moreover, multivariate analysis revealed no other significant contributing factors.

This study has several limitations, including its two-center, retrospective design, the lack of relapse-free survival assessment, and a relatively short follow-up period. In addition, the number of evaluable patients decreased at later response time points, particularly at the 12-month assessment, as patients who were refractory to high-dose dexamethasone (HDD) or who initially responded but subsequently relapsed and required second-line therapy were excluded from further response evaluations to avoid confounding effects from additional treatment lines. Although this approach allowed for a more isolated assessment of the impact of body mass index (BMI) on response to HDD, it may limit the generalizability of long-term response outcomes. Despite these limitations, the inclusion of a homogeneous cohort consisting exclusively of patients receiving HDD as first-line therapy, together with the relatively limited number of studies addressing the impact of BMI on treatment response in ITP, represents a notable strengssth of the present study. Prospective, randomized studies are warranted to further clarify the effect of BMI on treatment response.

## Conclusion

In this cohort, BMI did not influence early response to high-dose dexamethasone, although patients with BMI < 30 were more likely to achieve complete response at 12 months, while those with BMI ≥ 30 more commonly showed partial response. No demographic or clinical parameter consistently predicted treatment outcomes across follow-up points, and early associations lost significance in multivariable analyses. These findings support the continued use of a uniform 40 mg/day HDD regimen regardless of BMI, while suggesting that obesity may exert a delayed impact on long-term response. There remains a need for larger, prospective studies incorporating pharmacokinetic assessments to clarify the underlying mechanisms and to determine whether obesity-related factors should be integrated into individualized treatment strategies.

## Data Availability

The datasets generated and analyzed during the current study are available from the corresponding author on reasonable request.
